# Significance of thymidylate synthase expression for resistance to pemetrexed in pulmonary adenocarcinoma

**DOI:** 10.3892/ol.2013.1688

**Published:** 2013-11-19

**Authors:** MIN YANG, WEI-FEI FAN, XIAO-LIN PU, FU-YIN LIU, LI-JUAN MENG, JUN WANG

**Affiliations:** Department of Oncology, Jiangsu Province Geriatric Institute, Nanjing, Jiangsu 210009, P.R. China

**Keywords:** non-small cell lung cancer, pemetrexed, resistance, thymidylate synthase, reduced folate carrier, folypoly-γ-glutamate synthetase

## Abstract

Pemetrexed (PEM), a multi-targeted antifolate, has promising clinical activity in non-squamous non-small cell lung cancer. However, the majority of patients eventually acquire resistance to PEM. To evaluate the resistant mechanisms, the A549 lung adenocarcinoma cell line was exposed to stepwise increasing PEM concentrations of 1.6, 6.4 and 16 μM to establish three PEM-resistant lung cancer cell lines, A549/PEM-1.6, -6.4 and -16. Growth inhibition was determined by the 3-[4,5-dimethylthiazol-2-yl]-2,5-diphenyltetrazolium bromide assay. Expression of the genes encoding thymidylate synthase (TS), reduced folate carrier (RFC) and folypoly-γ-glutamate synthetase (FPGS) were analyzed by quantitative real-time reverse transcription polymerase chain reaction. The three A549 cell lines showed more resistance to PEM (3.7-, 17.3^−^ and 38.0-fold, respectively) compared with that of the parental cell line, which also showed cross-resistance to cisplatin, but not to docetaxel, vinorelbine and 5-Fluorouracil (5-FU). TS gene expression was significantly increased in three PEM-resistant cells, relative to that of the parental cells, in a PEM dose-dependent manner. Knockdown of TS expression with siRNA enhanced the cytotoxicity of PEM in A549/PEM-16 cells. By contrast, the levels of RFC and FPGS gene expression in A549/PEM-1.6 and -6.4 cells were significantly decreased, whereas the levels of the two genes were restored in A549/PEM-16 cells. In summary, PEM-resistant A549 cells remained sensitive to docetaxel, vinorelbine and 5-FU. TS expression appeared to be associated with resistance to PEM, which may be a predictive marker for PEM sensitivity in lung adenocarcinoma.

## Introduction

Lung cancer is the leading cause of cancer-related mortality worldwide, with ~226,160 new cases and ~160,340 mortalities predicted in 2012 in the United States (1). Non-small cell lung cancer (NSCLC) is a heterogeneous aggregate of histologies, including squamous cell carcinoma, adenocarcinoma and large cell carcinoma, and represents ~80–85% of all types of lung cancer (2). Despite the public awareness of NSCLC and increasing use of screening techniques, the majority of patients are likely to have advanced-stage non-operable disease at the time of diagnosis. Therefore, chemotherapy is often the first-line treatment for such patients.

Progress has been made in the treatment of advanced NSCLC during the past decade (3). The results of four previous multicenter randomized clinical trials evaluating the newer cytotoxic agents, alone or in combination with platinum-based chemotherapy, were shown to prolong survival, relieve symptoms in the majority of cases and improve patient quality of life (4–7). It is clear from these studies that no single regimen demonstrated a significant superiority compared with any other combination. However, in the last three years, important advances have been achieved in the treatment of advanced NSCLC (8). Previous results arising from the availability of pemetrexed (PEM) show that histology represents an important variable in decision making (9).

PEM is a novel, multi-targeted antifolate and its primary mechanism of action is to inhibit at least three different enzymes in the folate pathway: thymidylate synthase (TS), dihydrofolate reductase and glycinamide ribonucleotide formyltransferase (10). These enzymes are involved in the synthesis of nucleotides and, therefore, inhibition ultimately hinders RNA and DNA synthesis. During the process, the primary vehicle for the uptake of PEM is reduced folate carrier (RFC), which is retained in cells as polyglutamates, a process catalyzed by folypoly-γ-glutamate synthetase (FPGS). Polyglutamation results in an increased intracellular drug concentration and cytotoxicity (11).

In chemotherapy-naive patients with advanced NSCLC, combination chemotherapy with PEM and cisplatin has an efficacy similar to that of gemcitabine and cisplatin, which has been the standard first-line treatment for patients with advanced NSCLC, with improved tolerability. The median overall survival time (MST) was 10.3 months in the two arms (12). However, a pre-planned analysis of this trial for the histological subtype of NSCLC reported that adenocarcinoma patients have a higher MST on cisplatin/PEM compared with cisplatin/gemcitabine (12.6, vs. 10.9 months, respectively; P=0.03) (9). PEM produced similar results and had an improved tolerance compared with that of docetaxel in advanced NSCLC patients following the failure of one prior chemotherapy regimen in a phase III trial (13), with an MST of 8.3 versus 7.9 months, respectively. No significant difference was identified in the outcome or toxicity between elderly and younger patients (14). Thus, the majority of patients acquired resistance to PEM between 2 and 5 months. Therefore, in the current study, PEM-resistant lung adenocarcinoma cell lines were established to further understand the resistance mechanisms.

## Materials and methods

### Cell lines and chemicals

A549 cells were purchased from the American Type Culture Collection (Manassas, VA, USA), which were cultured in RPMI-1640 medium supplemented with 10% fetal bovine serum, penicillin G (100 U/ml) and streptomycin (100 μg/ml) in a humidified chamber (37ºC, 5% CO_2_). To observe the various mechanisms according to the degree of resistance, the A549 cell line was continuously exposed to stepwise increasing PEM concentrations of up to 1.6 μM for 5 months, 6.4 μM for 7 months and 16 μM for 10 months, which resulted in the following three PEM-resistant sublines: A549/PEM-1.6, -6.4 and -16. A549/PEM-1.6 cells were cultured in 1.6 μM PEM, A549/PEM-6.4 in 6.4 μM PEM and A549/PEM-16 in 16 μM PEM. PEM was obtained from Eli Lilly and Company (Indianapolis, IN, USA), docetaxel from Sanofi S.A (Paris, France), cisplatin and vinorelbine from Qilu Pharmaceutical Co., Ltd. (Shandong, China), 5-Fluorouracil (5-FU) from Xudong Haipu Pharmaceutical Co., Ltd. (Shanghai, China) and methotrexate (MTX) from Hengrui Medicine Co., Ltd. (Jiangsu, China). 3-(4,5-Dimethyl thiazol-2-yl)-2,5-diphenyltetrazolium bromide (MTT) was purchased from Sigma-Aldrich (St. Louis, MO, USA).

### Growth inhibition assay

Growth inhibition was evaluated using MTT assay, which measures the mitochondrial activity of viable cells. Cells were plated in flat bottom 96-well plates (Greiner Bio-One GmbH, Frickenhausen, Germany), with seeding densities of 2,000 cells per well for A549 and its sublines and allowed to attach for 24 h. Subsequently, cells were treated with RPMI-PBS containing serial dilutions of each chemotherapeutic agent for 96 h in a humidified chamber (37ºC, 5% CO_2_). Following treatment, the medium was removed and cells were incubated for 4 h at 37ºC in 50 μl per well of MTT solution (final concentration, 0.42 mg ml-1). Formazan crystals that had formed were dissolved in 150 μl dimethyl sulfoxide per well and the absorbance was measured at 540 nm using a spectrophotometric microplate reader (iMARK; Bio-Rad, Hercules, CA, USA). Radiosensitivity was also determined using the MTT assay. Instead of adding chemotherapeutic drugs, cells in a 96-well microplate were irradiated at doses of 0 (control), 4 or 8 Gy (room temperature; linear accelerator; URA, Antwerp, Belgium). Following 96 h incubation at 37ºC in a humidified chamber with 5% CO_2_, the cells were assayed as abovementioned.

The drug concentration required to inhibit the growth of tumor cells by 50% (IC_50_) was calculated by plotting the logarithm of the drug concentration versus the percentage of surviving cells. Each assay was performed in quadruplicate at least three times and the mean was calculated.

A growth rate of the cells was also determined by MTT assay and cells growing in exponential phase were plated in 48-well plates. The doubling time of each cell line was estimated from the duration of cell increment determined by measuring the mean absorbance of eight wells for seven successive days.

### Total RNA extraction and quantitative real-time reverse transcription-polymerase chain reaction (qPCR)

Total RNA was extracted from cells using a TRI Reagent kit (Molecular Research Center, Inc., Cincinnati, OH, USA) according to the manufacturer’s instructions. First-strand cDNA was synthesized using 1 μg of total RNA in a 20 μl RT reaction mixture containing 4 μl of 5X RT buffer (Gibco-BRL, Carlsbad, CA, USA), 2 μl DTT (100 mM), 4 μl dNTP (2.5 nM) and 1 μl superscript II RNase H reverse transcriptase (Gibco-BRL).

qPCR was performed using an ABI PRISM 7700 Sequence Detection system (Applied Biosystems, Inc., Foster City, CA, USA). The protocol was as follows: 50ºC for 2 min and 95ºC for 10 min, followed by 50 cycles at 95ºC for 15 sec and 60ºC for 2 min. The mRNA levels were normalized using GAPDH expression. The reaction mix consisted of 12.5 μl SYBR Green master mix (Applied Biosystems, Inc.) and 2.5 μl of forward and reverse primers for target gene (Table I) or 2.5 μl of the primer pair for GAPDH, 5 μl of each cDNA sample and 2.5 μl ddH_2_O.

### Transfection and siRNA experiments

A549/PEM-16 cells (1×10^6^) were transfected with siRNA oligonucleotides by X-tremeGENE siRNA transfection Reagent (Roche Diagnostics GmbH, Mannheim, Germany) according to the manufacturer’s instructions. Following 24 h, total RNA was extracted or the cells were cultured at a density of 5,000 per well in 96-well plates for 2 h. Following the addition of stepwise dilutions of PEM, the cultures were incubated at 37ºC for 48 h to assess cell viability. At the end of the culture period, 20 μl MTS solution was added followed by an additional 4 h incubation, prior to measuring the absorbance at 490 nm using an ELISA plate reader. The siRNA oligonucleotides for TS (predesigned siRNA; ID 116928) and negative control siRNA (silence negative control 1 siRNA) were purchased from Ambion (Carlsbad, CA, USA).

### Statistical analysis

Statistical significance was determined using one-way analysis of variance. P<0.05 was considered to indicate a statistically significant difference using two-sided analysis.

## Results

### Establishment of three PEM-resistant lung cancer sublines

**Figure 1:**
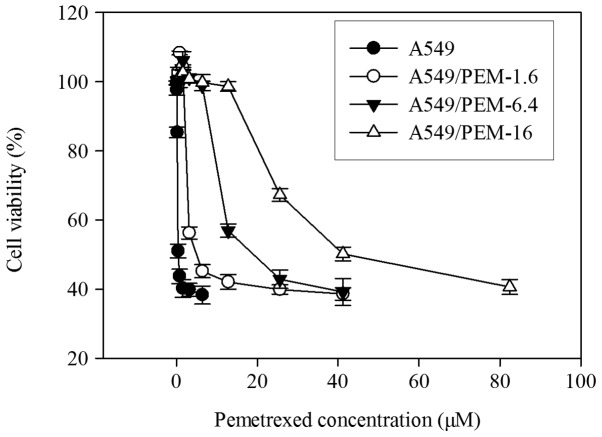
Dose response curves of A549 and A549/PEM-1.6, -6.4 and -16 cells to PEM. PEM, pemetrexed.

To investigate the determinants of acquired resistance to PEM in lung adenocarcinoma, three PEM-resistant cell lines, A549/PEM-1.6, -6.4 and -16, were established (Fig. 1). The IC_50_ values of PEM for A549/PEM-1.6, -6.4 and -16 cells were ~5.0, 23.4 and 51.5 μM, respectively and the cells were more resistant by ~3-, 17- and 37-fold, respectively, relative to A549 cells (Table II). The three sublines were significantly more resistant than their parental cell lines to PEM (all P<0.05). The doubling times of each cell were as follows: A549, 18.9 h; A549/PEM-1.6, 21.3 h; A549/PEM-6.4, 20.7 h; and A549/PEM-16, 19.1 h. The growth rate of these sublines did not change (P>0.05).

Cross-resistant patterns were also observed for these three cell lines (Table II). All three PEM-resistant sublines exhibited cross resistance to cisplatin, but not to docetaxel, vinorelbine and 5-FU, and also remained sensitive to MTX, a mother compound of PEM.

### Radiosensitivity

**Figure 2:**
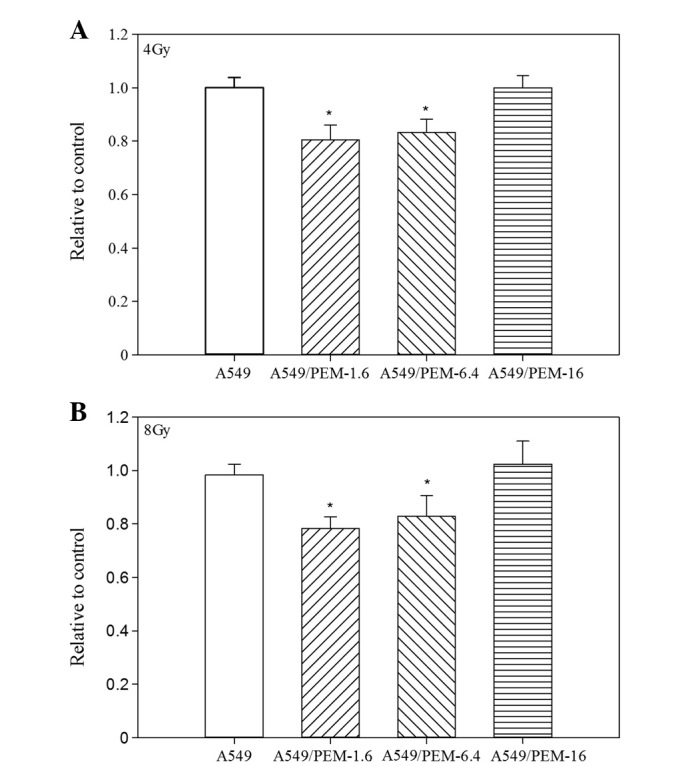
Sensitivity to irradiation was determined using MTT assays in A549 and A549/PEM-1.6, -6.4 and -16 cells at (A) 4 and (B) 8 Gy. The surviving fraction was evaluated using the ratio of IC_50_ of irradiated cells compared with that of non-irradiated cells. MTT, 3-(4,5-dimethylthiazol-2-yl)-2,5-diphenyltetrazolium bromide; PEM, pemetrexed; IC_50_, 50% inhibitory concentration.

PEM-resistant subline cells revealed a distinctive sensitivity to irradiation. A549/PEM-1.6 and -6.4 cells showed more sensitivity than the parental A549 cells to irradiation. However, highly PEM-resistant A549/PEM-16 cells did not (Fig. 2A and 2B).

### Expression levels of TS, RFC and FPGS genes in three PEM-resistant sublines

**Figure 3:**
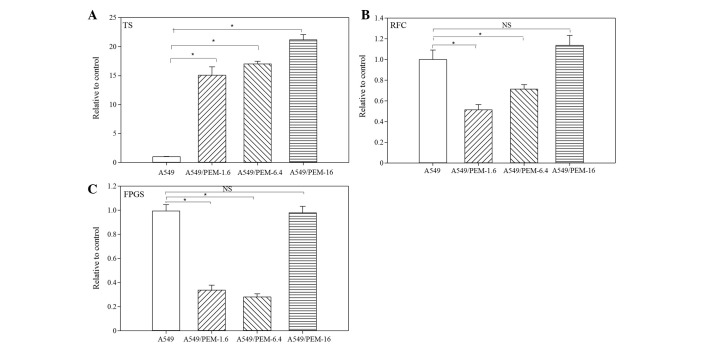
Expression levels of TS, RFC and FPGS genes in A549 and A549/PEM-1.6, -6.4 and -16 cells were determined by real-time reverse transcription-polymerase chain reaction from three independent experiments. ^*^P<0.05. NS, no significant difference; TS, thymidylate synthase; RFC, reduced folate carrier; FPGS, folypoly-γ-glutamate synthetase.

qPCR was performed to compare the expression levels of TS, RFC and FPGS in three PEM-resistant lung adenocarcinoma cells with those of the parental A549 cells. Compared with A549 cells, the levels of TS gene expression were significantly increased in A549/PEM-1.6 (15.1-fold; P<0.05), -6.4 cells (17.0-fold; P<0.05)and -16 (21.2-fold; P<0.05) cells (Fig. 3A). TS gene expression increased with increasing stepwise concentrations of PEM. RFC gene expression was decreased in A549/PEM-1.6 (51.3%; P<0.05) and -6.4 (71.3%; P<0.05) cells, but restored in A549/PEM-16 cells to levels similar to those of the parental A549 cells (Fig. 3B). FPGS gene expression was diminished in A549/PEM-1.6 and -6.4 cells (34.3 and 28.3%, respectively; P<0.05), but not in A549/PEM-16 cells (Fig. 3C).

### Effect of TS siRNA

**Figure 4:**
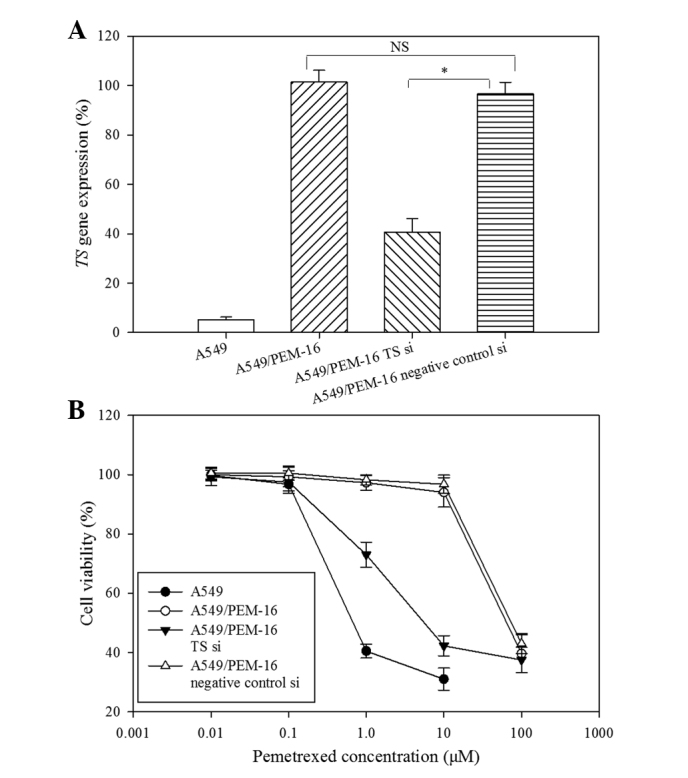
Modification of PEM cytotoxicity using TS siRNA. (A) Expression of TS gene and (B) cytotoxicity of 0.01, 0.1, 1, 10 and 100 μM PEM in A549/PEM-16 cells transfected with TS siRNA, in negative control siRNA and non-transfected A549/PEM-16 cells. ^*^P<0.05. PEM, pemetrexed; TS, thymidylate synthase; NS, no significant difference.

Considering the importance of TS overexpression for acquired resistance to PEM, A549/PEM-16 cells were transfected with TS siRNA to investigate whether modification of TS gene expression may alter PEM cytotoxicity. At 24 h following transfection, total RNA was extracted and TS gene expression was measured by real-time RT-PCR. Relative to A549/PEM-16 transfected with negative-control siRNA, the expression of the TS gene was significantly diminished by ~41% in cells treated with TS siRNA (P<0.05) and was not changed in non-transfected cells (Fig. 4A). At 72 h following transfection, cell viability was assessed using MTS assays, which showed that the cytotoxicity of PEM in A549/PEM-1.6 cells transfected with TS siRNA was greatly enhanced compared with cells transfected with negative-control siRNA (Fig. 4B). Therefore, decreased TS gene expression altered the sensitivity of PEM.

## Discussion

Three PEM-resistant lung adenocarcinoma cell lines were established with three different PEM concentrations, which remained sensitive to 5-FU, docetaxel and vinorelbine, however, all cell lines showed resistance to cisplatin.

TS has a central role in DNA biosynthesis and tumor biology and is the target of antifolate agents, such as 5-FU. The acute induction of TS has also been verified as one of several mechanisms of acquired resistance to 5-FU, since TS is stably bound to FdUMP and no longer has the ability to bind to its mRNA and suppress its own translation, which results in increased TS protein expression (15). Notably, incubation of TS with PEM also significantly impairs its ability to interact with TS mRNA (15). Therefore, acute induction of TS expression may be important for acquired resistance to PEM in the same manner as 5-FU. In the present study, the expression of TS mRNA was significantly increased in A549/PEM-1.6, -6.4 and -16 cells compared with A549 cells, which was consistent with previous studies (16–19). In addition, following transfection with TS siRNA, the expression of the TS gene in A549/PEM-16 cells was diminished significantly and sensitivity to PEM was restored.

High TS expression in uterine cervical cancer cells has been reported to induce resistance to radiation, which has been explained by the suppression of p53 expression or promotion of DNA repair via TS increment (20). However, in the current study, no correlation between high TS expression and radiation was detected. By contrast, A549/PEM-1.6 and -6.4 cells showed increased sensitivity compared with parental cells to irradiation. Therefore, further investigation to clarify the correlation between TS levels and resistance to radiation is required. The results of the present study also indicated that PEM-resistant patients with locally advanced NSCLC in clinical settings may remain sensitive to irradiation, but receive thoracic radiotherapy. However, this is likely to be confirmed by future clinical trials.

RFC and FPGS activities may also be a determinant of PEM cytotoxicity (21,22). PEM utilizes RFC for entry into cells and then requires polyglutamation by FPGS to inhibit various target enzymes maximally. Therefore, decreased expression of RFC and/or FPGS may be associated with resistance to PEM, which has been determined in an L1210 murine leukemia cell line (23) and colon cell line (21). In the present study, RFC and FPGS gene expression in A549/PEM-1.6 and -6.4 cells was significantly decreased, while RFC and FPGS gene expression in A549/PEM-16 cells was restored to levels similar to those observed in parental A549 cells. Therefore, acquired resistance to PEM may result from the reduction of the intracellular concentration of PEM due to decreased levels of RFC gene expression and/or inhibition of polyglutamation due to decreased levels of FPGS gene expression in low (1.6 μM) or medium (6.4 μM) concentrations. However, in high (16 μM) concentrations, the determinant of acquired resistance to PEM is different, which may particularly depend on high TS gene expression. Although the determinants of acquired resistance to PEM may be altered by PEM concentration, TS overexpression may be one of the major determinants.

Three PEM-resistant lung adenocarcinoma cell lines were established, which remained sensitive to 5-FU, docetaxel and vinorelbine. It has been proposed that TS overexpression may be one of the major determinants of acquired resistance to PEM in lung adenocarcinoma, although, its interaction with other genes, such as RFC and FPGS, may also be important. In conclusion, we hypothesize that the level of TS gene expression may predict drug sensitivity to PEM. Therefore, examination of the correlation between TS gene expression and sensitivity to PEM in patients of lung adenocarcinoma is predicted.

## Figures and Tables

**Table I:** Primers used in PCR.

Protein	Primer
TS	
Forward	CAC ACT TTG GGA GAT GCA CAT ATT
Reverse	TTC GAA GAA TCC TGA GCT TTG G
FPGS	
Forward	CTA TGC CGT CTT CTG CCC TAA C
Reverse	ACC TGG TCC AGT GTC ACT GTG A
RFC	
Forward	CGT CAA GAC CAT CAT CAC TTT CA
Reverse	CAG GAT CAG GAA GTA CAC GGA GTA T

TS, thymidylate synthase; FPGS, folypoly-γ-glutamate synthetase; RFC, reduced folate carrier.

**Table II:** Drug sensitivity in the parental A549 cell line and PEM-resistant sublines.

	IC_50_ (95% CI), μM			
	
Drug	A549	A549/PEM-1.6	A549/PEM-6.4	A549/PEM-16
PEM	1.35 (0.93–2.12)	5.03 (2.16–7.82)	23.39 (17.86–32.52)	51.45 (43.03–64.55)
RR		3.7*	17.3*	38.0*
CDDP	1.11 (0.85–1.47)	1.78 (1.51–2.16)	1.84 (1.53–2.30)	1.89 (1.54–2.45)
RR		1.6*	1.7*	1.7*
DOC	0.0013 (0.0009–0.0019)	0.0013 (0.0009–0.0020)	0.0014 (0.0010–0.0021)	0.0011 (0.0008–0.0017)
RR		1.0	1.1	0.8
VNR	0.018 (0.015–0.024)	0.019 (0.015–0.025)	0.016 (0.013–0.022)	0.017 (0.013–0.024)
RR		1.1	0.9	0.9
5-FU	1.85 (1.44–2.61)	1.67 (1.24–2.51)	1.62 (1.16–2.58)	1.70 (1.27–2.52)
RR		0.9	0.9	0.9
MTX	0.021 (0.016–0.031)	0.023 (0.017–0.036)	0.025 (0.018–0.039)	0.026 (0.020–0.040)
RR		1.1	1.2	1.2

Sensitivity to the drugs was evaluated by MTT assay after 96 h. Values are means of at least three independent experiments and the 95% CIs were calculated. RR was calculated as follows: RR = IC_50_ in the resistant subline/IC_50_ in the parental subline. MTT, 3-(4,5-Dimethylthiazol-2-yl)-2,5-Diphenyltetrazolium bromide; RR, resistance rate; IC_50_, 50% inhibitory concentration; 95% CI, 95% confidence interval; PEM, pemetrexed; CDDP, cisplatin; DOC, docetaxl; VNR, vinorelbine; 5-FU, 5-Fluorouracil; MTX, methotrexate.

* P<0.05, vs. the parental cell line.
